# Bilateral Intratesticular Spermatoceles in a Critically Endangered Dama Gazelle (*Nanger dama mhorr*)

**DOI:** 10.1155/2020/8810770

**Published:** 2020-07-16

**Authors:** Riley E. Thompson, Holly J. Haefele, Daniel P. O'Neil, Budhan S. Pukazhenthi

**Affiliations:** ^1^Center for Species Survival, Smithsonian Conservation Biology Institute, Front Royal, VA 22630, USA; ^2^Department of Animal Health and Research, Fossil Rim Wildlife Center, Glen Rose, TX 76043, USA; ^3^Pedernales Veterinary Center, Fredericksburg, TX 78624, USA

## Abstract

Dama gazelles (*Nanger dama mhorr*) are considered critically endangered by the IUCN Red List of Threatened Species since the wild population is comprised of fewer than 200 animals. Several zoos in Europe and some private ranches in the US (Texas) maintain this species in captivity as a hedge against extinction. A routine reproductive exam on an adult male revealed bilateral cysts in the testicular parenchyma. The fluid in the cysts contained copious, moderately progressive motile spermatozoa. Following examination, the gazelle was diagnosed with bilateral intratesticular spermatoceles. Electroejaculation yielded 1.5 ml of semen containing 387 × 10^6^ total sperm with 50% motility and 45% morphologically normal sperm. The spermatoceles did not appear to cause pain or dysfunction, so no treatment was performed at this time. Since fewer than 10 animals are managed in captivity in the US, no intervention (castration/hemicastration) was considered.

## 1. Introduction

Spermatoceles and/or spermatic granulomas, a sperm-filled cystic structure similar to a spermatocele resulting in a granulomatous reaction from sperm extravasation caused by necrosis [1], have been reported in men, rams, and stallions [2–5]. Most sperm-filled cysts are localized to the caput or cauda epididymis [6]. However, spermatoceles have also been reported in the testicular parenchyma of men and rams [7]. To our knowledge, there have been no reports of spermatoceles, epididymal or intratesticular, in gazelles. This case reveals an important differential for anechoic, cystic structures seen on testicular ultrasounds of wildlife species.

## 2. Case Presentation

**Figure 1:**
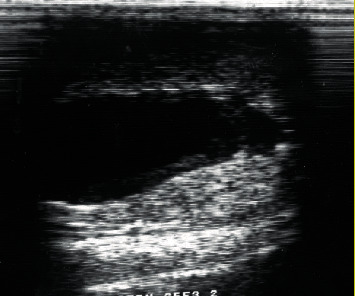
Ultrasound image of intratesticular spermatocele in a Dama gazelle.

**Figure 2:**
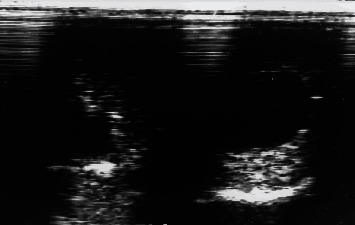
Ultrasound image of bilateral intratesticular spermatoceles in a Dama gazelle. Spermatocele appears as fluid-filled (anechoic) cystic structures.

An adult male Dama gazelle (*Nanger dama mhorr)* was presented for routine reproductive examination. Animal was anesthetized (via dart; IM) using a combination of butorphanol tartrate (0.5 mg/kg), azaperone tartrate (0.2 mg/kg), and medetomidine hydrochloride (0.2 mg/kg; BAM™, ZooPharm, Laramie, Wyoming, USA). Anesthesia was supplemented with ketamine hydrochloride (1.0 mg/kg; IV) and propofol (0.5 mg/kg; IV) to maintain an optimal anesthetic plane. Animal was subjected to a physical examination, testicular measurements, an ultrasound assessment of testes (5 MHz linear probe), and semen collection by electroejaculation [8]. Testicular ultrasound revealed well-demarcated, round, anechoic structures in the testicular parenchyma of both testes (Figures 1 and 2). Electroejaculation yielded 1.5 ml semen containing 387 × 10^6^ sperm with ~50% total sperm motility (assessed by subjective microscopic evaluation) and 45% morphologically normal sperm (assessed using a phase contrast microscope at 1,000x magnification). Aspiration of the anechoic structures using a sterile hypodermic needle (20 G; 1.5 inch) yielded a cloudy fluid with large numbers of spermatozoa with moderate progressive motility. Anesthesia was reversed using atipamezole (1.0 mg/kg) and naltrexone (0.3 mg/kg) both administered via IM injections. No treatment was performed due to lack of pain and/or testicular dysfunction. Furthermore, since Dama gazelles are critically endangered with only 10 individuals of this subspecies managed in captivity in the US, castration or hemicastration was ruled out.

## 3. Discussion

A spermatocele is a benign, sperm-filled cyst of unknown etiology [9], and a spermatic granuloma is a sperm-filled cystic structure similar to spermatoceles resulting from a granulomatous reaction due to sperm extravasation caused by necrosis [1]. The lesion in the present case was categorized as a spermatocele rather than a spermatic granuloma because the cysts were bilateral and there was no history of trauma or testicular neoplasia that could have given rise to tissue necrosis. *Brucella ovis* has been associated with spermatoceles and spermatic granulomas in rams [10], but there is no medical or herd history to suggest that this lesion is associated with brucellosis in this case. No information has been previously reported on the incidence of spermatoceles in Dama gazelles. Spermatoceles have been reported in rams, and most are localized to the caput or cauda region of the epididymis [6], though an intratesticular spermatocele has previously been reported in a Merino ram [7]. While most spermatoceles are small and considered incidental, if spermatoceles grow too large, they have been shown to cause pain in men [11]. In the current case, the spermatocele did not appear to cause pain or affect sperm output.

Other causes of round, anechoic structures in the testicular parenchyma include simple cysts, intratesticular abscess, and intratesticular infarction [12]. Cystic transformation of the rete testis does not typically result in a regular, round structure [12]. To distinguish between a spermatocele and other causes of round, anechoic structures in the testicular parenchyma, a needle aspirate must be performed and examined grossly and microscopically.

Three other Dama gazelles evaluated at the same time as this individual produced 1.5-4.6 ml ejaculates containing 0.18 − 2.11 × 10^9^ total sperm with 50-90% total sperm motility and 24-93% morphologically normal sperm. These results were comparable to the individual with the spermatoceles and to previous reports [13, 14]. The wide variation observed in ejaculate quality among animals and the small sample size do not permit a thorough assessment of the impact of spermatocele in Dama gazelles. Hence, further studies are warranted.
